# In Vitro Assessment of Early Bacterial Activity on Micro/Nanostructured Ti6Al4V Surfaces

**DOI:** 10.3390/molecules22050832

**Published:** 2017-05-18

**Authors:** Benjamin Valdez-Salas, Ernesto Beltrán-Partida, Sandra Castillo-Uribe, Mario Curiel-Álvarez, Roumen Zlatev, Margarita Stoytcheva, Gisela Montero-Alpírez, Lidia Vargas-Osuna

**Affiliations:** 1 Instituto de Ingeniería, Departamento de Corrosión y Materiales, Universidad Autónoma de Baja California, Blvd. Benito Juárez y Calle de la Normal s/n, Mexicali C.P., 21040 Baja California, Mexico; beltrane@uabc.edu.mx (E.B.-P.); sandra.castillo@uabc.edu.mx (S.C.-U.); mcuriel@uabc.edu.mx (M.C.-Á.); roumen@uabc.edu.mx (R.Z.); margarita.stoytcheva@uabc.edu.mx (M.S.); gmontero@uabc.edu.mx (G.M.-A.); 2 Facultad de Odontología Mexicali, Universidad Autónoma de Baja California, Av. Zotoluca y Chinampas, s/n, Mexicali C.P., 21280 Baja California, Mexico; 3 Facultad de Ingeniería, Universidad Autónoma de Baja California, Blvd. Benito Juárez y Calle de la Normal s/n, Mexicali C.P., 21040 Baja California, Mexico; lidia.vargas@uabc.edu.mx

**Keywords:** bacterial adhesion, nanostructure, titanium implant, Gram-positive, Gram-negative, biomaterial infections, TiO_2_ nanotubes

## Abstract

It is imperative to understand and systematically compare the initial interactions between bacteria genre and surface properties. Thus, we fabricated a flat, anodized with 80 nm TiO_2_ nanotubes (NTs), and a rough Ti6Al4V surface. The materials were characterized using field-emission scanning electron microscopy (FE-SEM), energy dispersive X-ray spectroscopy (EDX) and atomic force microscopy (AFM). We cultured in vitro *Staphylococcus epidermidis* (*S. epidermidis*) and *Pseudomonas aeruginosa* (*P. aeruginosa*) to evaluate the bacterial-surface behavior by FE-SEM and viability calculation. In addition, the initial effects of human osteoblasts were tested on the materials. Gram-negative bacteria showed promoted adherence and viability over the flat and rough surface, while NTs displayed opposite activity with altered morphology. Gram-positive bacteria illustrated similar cellular architecture over the surfaces but with promoted surface adhesion bonds on the flat alloy. Rough surfaces supported *S. epidermidis* viability, whilst NTs exhibited lower vitality. NTs advocated promoted better osteoblast organization with enhanced vitality. Gram-positive bacteria suggested preferred adhesion capability over flat and carbon-rich surfaces. Gram-negative bacteria were strongly disturbed by NTs but largely stimulated by flat and rough materials. Our work proposed that the chemical profile of the material surface and the bacterial cell wall characteristics might play an important role in the bacteria-surface interactions.

## 1. Introduction

Titanium (Ti) and Ti-based alloys (such as Ti6Al4V) are amongst the most used metallic materials for different bone replacements [1,2]. As Ti present important attributes such as low toxicity, corrosion resistance (in physiological environments), proper mechanical properties and, more importantly, excellent biocompatibility (mainly when implanted in healthy patients), Ti is considered the most important option for orthopedic implantable devices [3].

Despite the fact that orthopedic surgical procedures are executed under strict sterilization techniques, implant-associated infections (i.e., peri-prosthetic infections) are still present and are considered one of the main challenges in orthopedic surgery [4,5,6]. A rising approach for the molecular control of bacteria adhesion and consequent biofilm formation (which is considered the main bacterial organization associated with implant infections) is the synthesis of nanostructured materials with defined morphology and homogenous topographies [7,8,9]. For example, Puckett et al. [7] suggested that nanostructured and nanorougher Ti surfaces can control the adhesion of important orthopedic related bacteria such as *Staphylococcus aureus* (*S. aureus*), *S. epidermidis*, and *P. aeruginosa* (Gram-negative bacteria) by reducing the retention number of live bacteria and increasing the level of retained dead cells after 1 h of incubation. Moreover, Pérez-Jorge et al. [8] advocated decreased *S. aureus* and *S. epidermidis* (Gram-positive bacteria) colonization over nanoporous and nanotubular Ti6Al4V surfaces after comparing them to those of flat topographies, illustrating a similar behavior between the two bacterial models. However, these important works did not solidly analyze the impact of the morphological properties of bacteria; they did not highlight the bacteria genre properties at the surface-bacterial interactions and did not compare the modulatory effects of nanostructured surfaces versus those of a micro-rough surface. Thus, we hypothesized that the surface physicochemical features of a flat, rough, and nanostructured Ti6Al4V alloy and the bacterial genre (Gram-positive or negative) will promote different microbiological behaviors on a Ti6Al4V surface, suggesting that part of the bacterial adhesion capability will be modulated by the bacteria’s ability to produce adhesion-bonds at the nanoscale-bacterial interface with varied bacterial morphologies.

The present study evaluates and compares for the first time the bacterial-surface interaction at the micro- and nanoscale levels, as well as the adhesion and viability of *P. aeroginosa* and *S. epidermidis* (crucial models of orthopedic related biomaterial infections) onto a nanotubular (defined as NTs), microstructured (labeled as rough), and a smooth (indicated as Flat) Ti6Al4V alloy. The main objective was to describe the part of the mechanism involved in the initial bacterial behavior, the effect of Gram-positive and Gram-negative bacteria, and the protagonist role of the morphological/topographical properties of the material surface for bone implant applications. Moreover, the early trend of human osteoblast fashioning was interrogated as an endeavor to provide an important profile analysis of these medical imperative materials.

## 2. Results and Discussion

### 2.1. Ti6Al4V Surface Physicochemical Properties

**Figure 1:**
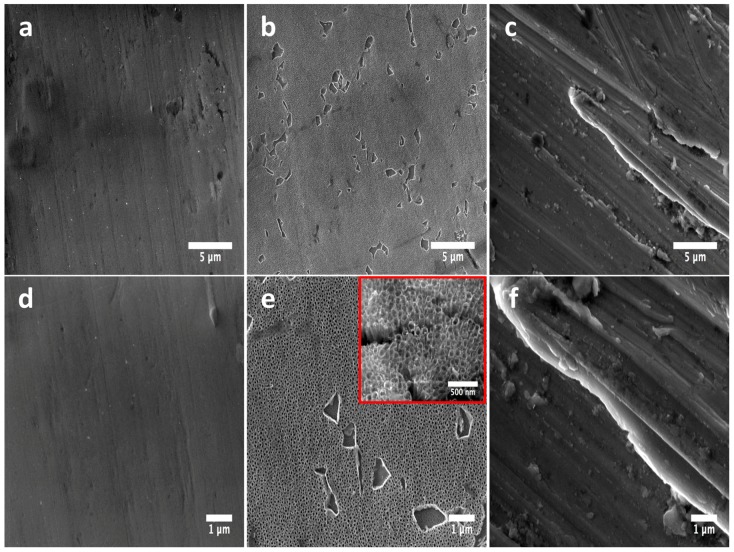
FE-SEM micrographs illustrating the surface morphology of the experimental materials: (**a**) Flat-Ti6Al4V surface showing a flat and smooth surface; (**b**) anodized 80 nm nanotubes (NTs) highlighting the homogenous formation of a nanostructured layer; (**c**) Rough-Ti6Al4V surface presenting irregular grooves among the material; (**d**) high zoom of the flat surface demonstrating the non-presence of a nanostructured surface; (**e**) high amplification of the NTs confirming the nanotubular homogeneity (inset represents a superior magnification, which described the nanotubular morphology); (**f**) high magnification of the rougher surface.

Field Emission Scanning Electron Microscopy (FE-SEM) clearly revealed the presence of a flat and smooth surface with slightly irregular surface properties (Figure 1a,d). Moreover, the anodized material illustrated the formation of well-defined 80 nm NTs on the surface, as represented in Figure 1b,e, following similar results as previously reported [10]. On the other hand, the rough alloy surface evidently showed an irregular surface with outcomes of surface imperfections (Figure 1c,f). Importantly, part of our experimental materials (Flat and Rough) are partially similar to those of some commercially available surgical Ti implants [11]. For example, the Brånemark^®^ Implant System (Manufactured by NobelBiocare AB S-402 26 Göteborg, Sweden) is fabricated by a machined technique, which generates clearly visible, well-defined, unidirectional structures, following a similar morphology to that observed with our flat material. Moreover, our rough surface is in concordance with the *3i* ICE Implant System (*3i* Implant Innovation Inc., Palm Beach Gardens, FL, USA), which showed similar surface imperfections on a rough polished surface, information that suggests that the biological behavior of our tested materials could be in line to those of commercial implantable systems.

Surface chemistry plays an important role in the control of the biological properties of biomaterials [12]. Thus attention was paid to the surface elemental composition, and the results presented in Table 1. Interestingly, the flat and rough surfaces present significantly differences in the oxygen content (not detectable on Flat and 5.98% on Rough) compared to the NTs (23.46%). This important difference could be related to the anodization process [10]. It is well known that anodization using fluoride electrolytes is able to strongly oxidize the surface of Ti-based materials, resulting in promoted levels of oxygen due to a thicker oxide layer formed over the surface. Moreover, our controlled anodization arrangement allowed the homogenous oxidation of the material surface with nanotubular morphology, information that it is in line with previous reports that also used fluoride [7,8]. On the other hand, the wettability parameter for the flat material is 70.02° ± 2.96° [13], for the NTs is 31.56° ± 2.62° [13], and for the rough surface is considered to be approximately 60.4° ± 5.8° [14]. The information suggests that the more hydrophilic material is the NTs, followed by the rough, and finally by the flat surface; however, with significant differences among them.

**Figure 2:**
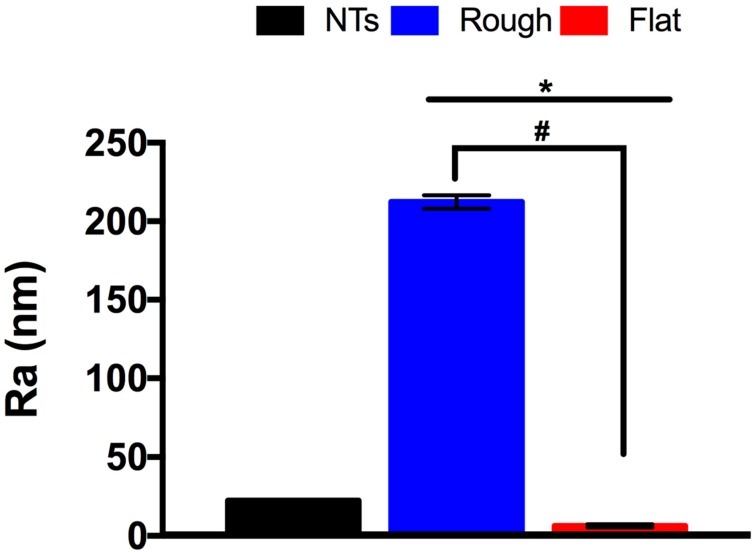
Analytical surface roughness comparison of the experimental substrates. * indicates significant differences between NTs and all the materials. # shows imperative changes among the rough and flat specimens.

The analytical surface roughness properties generated by the Atomic Force Microscopy (AFM) analysis strongly supports the information acquired by the FE-SEM examinations. As presented in Figure 2, the rough material showed an exuberant rougher surface after comparing it to the NTs and the flat surface. Moreover, the NTs also presented superior roughness to the flat polished material; information that is in line with the FE-SEM results and with previous works of anodized versus flat Ti surfaces [10,15].

### 2.2. Bacterial Activity of the Materials

**Figure 3:**
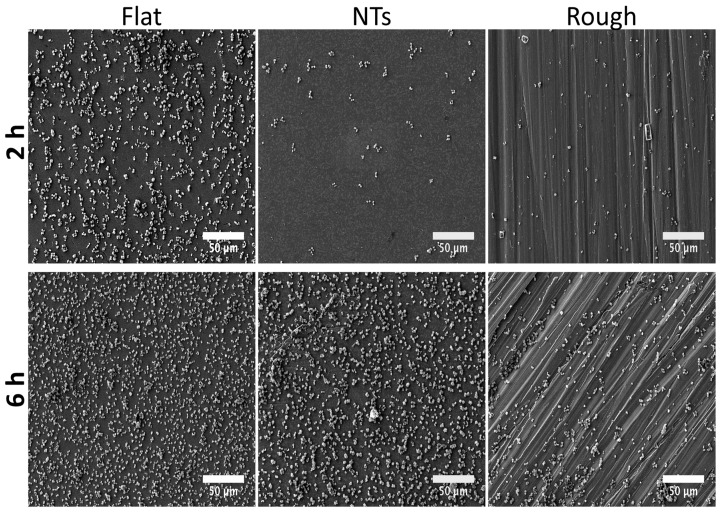
Initial and late colonization behavior of *S. epidermidis* over the experimental specimens as a function of time. A superior number of *S. epidermidis* can be highlighted over the flat surface at all incubation times.

*S. epidermidis* is an important Gram-positive bacteria that plays a vital role in orthopedic implant-related infections [6]. Therefore, taking into consideration this important issue, we evaluated its adhesion process at 2 and 6 h (Figure 3). At 2 h, a promoted adhesion rate can be observed over the flat surface, compared to the NTs and even with the rough material. This is surprising information, which suggests that a flat Ti6Al4V surface can stimulate faster *S. epidermidis* adhesion. After 6 h of incubation, we detected an increased amount of bacteria over all the work surfaces; however, of notable importance was a superior adhesion frequency over the flat material (with outcomes of biofilm formation) compared to the nanostructured and the rough material. On the other hand, a similar tendency was detected over the NTs and the rough-Ti6Al4V, information that is controversial to other reported studies [16], but its important to remain that flat polished surfaces are not a necessary option for avoiding bacterial adhesion.

**Figure 4:**
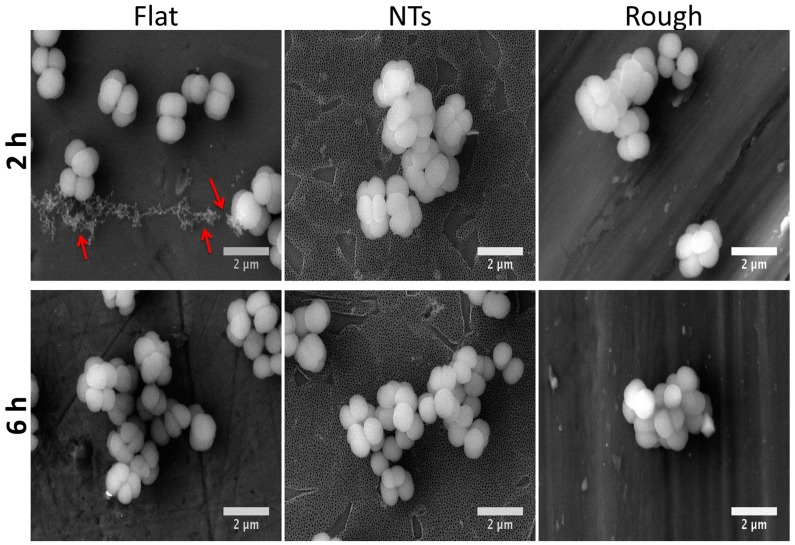
*S. epidermidis* organization onto the materials surfaces at 2 and 6 h of incubation. Note that the flat surface mainly stimulates the synthesis of exopolisacharides at 2 h of growth (red arrows).

**Figure 5:**
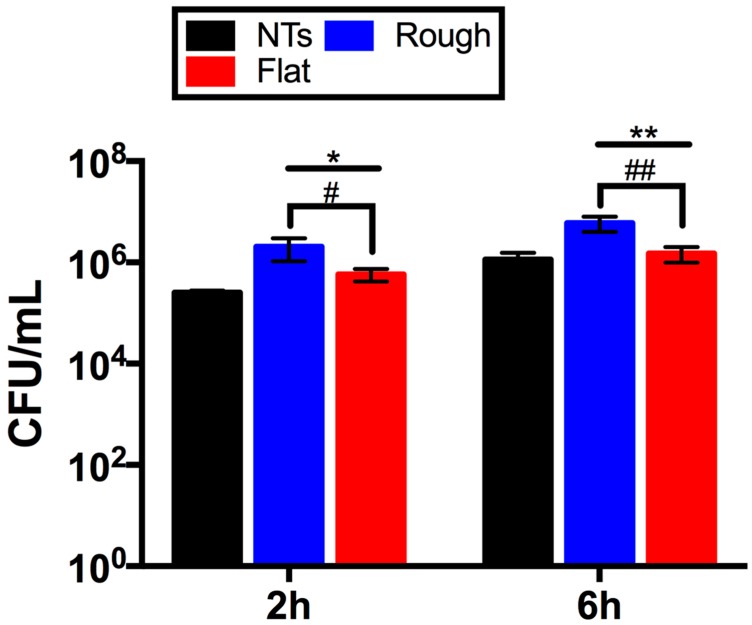
*S. epidermidis* viability onto the materials’ surfaces at 2 and 6 h of cultivation. * denotes significant differences between rough and flat materials versus NTs at 2 h. # symbolizes major changes in the flat and rough surfaces at 2 h. ** illustrates important variations between NTs and flat and rough specimens at 6 h. ## symbolize discrepancies for rough and flat samples at 6 h.

Bacterial morphology is of crucial importance for the characterization and interpretation of the possible involved mechanisms in cellular adhesion. Thus, in order to evaluate this important characteristic we applied a higher magnification to the adherent cells (Figure 4). The flat material presents similar bacterial morphology at both incubation times. Similarly, the rougher surfaces (NTs and Rough) showed parallel outcomes of cellular organization with a grape-like cluster arrangement. Moreover, the flat surface illustrated greater cell-cell adherence, suggesting initial biofilm formation; meanwhile, the NTs slightly advocate this organization. Additionally, significantly decreased staphylococci viability was detected over the NTs compared to the flat and rough materials at all incubation times (Figure 5). The NTs material probably illustrated decreased *S. epidermidis* adhesion and viability due to the low levels of fluoride (an antibacterial element) that are generated during the anodization process. As previously suggested for anodized nanostructured surfaces with higher levels of fluoride [8,17], the presence of F is capable of disrupting the adhesion process of bacteria, resulting in it being more difficult for bacteria to grow, as illustrated here. However, is important to highlight that our nanostructured material presents lower F content (2.71%) than those previously reported (4–6%) in Perez-Jorge et al. [8] and (4.76–13.59%) in Ercan et al. [17], which could suggest lower cellular toxicity. These important behaviors are in agreement with preceding works that evaluated and compared the structural capability of *S. epidermidis* over flat, nanostructured, and microstructured materials [18].

**Figure 6:**
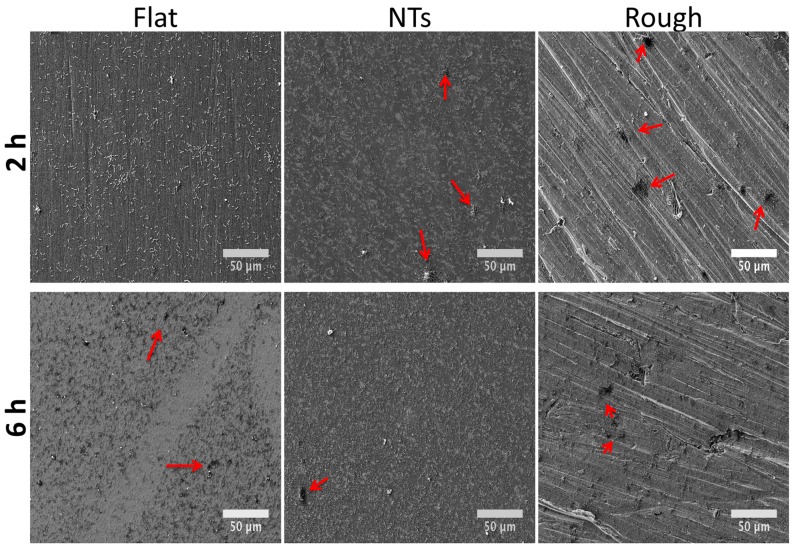
*P. aeruginosa* colonization over the materials at 2 and 6 h of growth. Red arrows symbolize the initial formation of bacterial colonies.

**Figure 7:**
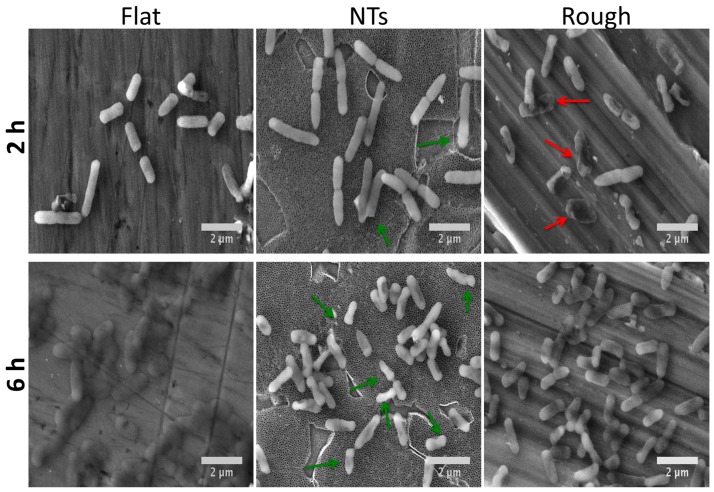
*P. aeruginosa* aggrupation on the materials at 2 and 6 h of growth. Red arrows symbolize aberrant bacilli morphology on the rough material. Green arrows highlight the deformed bacilli structure of the NTs.

Gram-negative bacteria (mainly *P. aeruginosa*) have been seriously involved in the microbiota related to orthopedic implant-associated infections [19]. Consequently, we evaluated the *P. aeruginosa* colonization activity in the experimental materials (Figure 6). The initial growing phase (2 h) showed important outcomes in colony organization on the rough surface and continued with an inferior tendency for the NTs and a poorer colony-forming capability on the flat material but with more consistent bacterial spreading. It is important to discuss that the promoted bacilli activity shown over the flat and rough experimental materials models evaluated here are consistent with previous works regarding the effect of surface roughness and the comparison of nanostructured morphologies versus microstructured materials [20,21]. The data suggest a greater affinity of *P. aeruginosa* with the rough and flat surface than the nanotubular one. This interesting trend could be explained in part by to the promoted carbon percentage detected on the flat and rough materials. As an elevated percentage of carbon in metallic materials was associated with an increased hydrophobicity [13,22] and *P. aeruginosa* has been suggested as a partially hydrophobic bacteria [23], it could be congruent to speculate that carbon-richer surfaces may be more related to attracting and promoting bacterial anchorage than hydrophilic surfaces. This information is consistent with the bacterial behavior and bacilli morphology (aberrant morphology mainly on NTs) (Figure 7) observed here.

**Figure 8:**
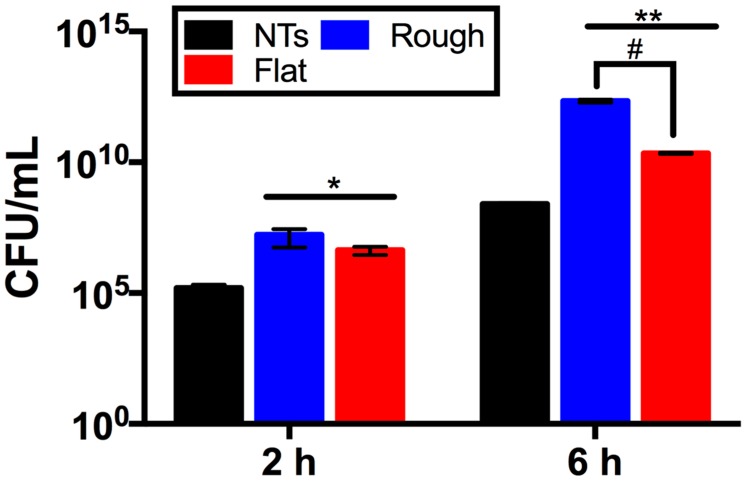
Analytical evaluation of *P. aeruginosa* viability over the experimental surfaces. * indicates significantly differences between NTs and the tested materials at 2 h. ** represents important differences comparing NTs and working surfaces at 6 h. # denotes critical changes between rough and flat materials at 6 h.

A crucial parameter required to evaluate and predict the bacterial behavior of biomaterials is the viable activity presented over the analyzed surfaces. Therefore, we characterized *P. aeruginosa* viability over the experimental surfaces (Figure 8). We detected that the NTs significantly decrease the viable number of colony forming units (CFU) after comparing them to the rough and flat materials at all incubation times. Moreover, similar behavior was detected after comparing the rough and flat surfaces (at 2 and 6 h), suggesting that the rougher material may allow more bacterial retention but with low outcomes of a robust cellular adhesion, as depicted in Figure 6 and Figure 7. Furthermore, as mentioned above we could suggest that the electrostatic attraction stimulated by the flat surface may be bigger (more hydrophobic) than that of the rough material, resulting in promoted viable cells (possibly by cell-cell bounding) over the rough surface but with decayed cellular adhesion, as observed here. However more molecular analyses such as the inhibition of bacterial receptors involved in the adhesion process, real time polymerase chain reaction (RT-PCR), western blot, and immunofluorescence assays are recommended in order to clarify those interesting interrogations.

**Figure 9:**
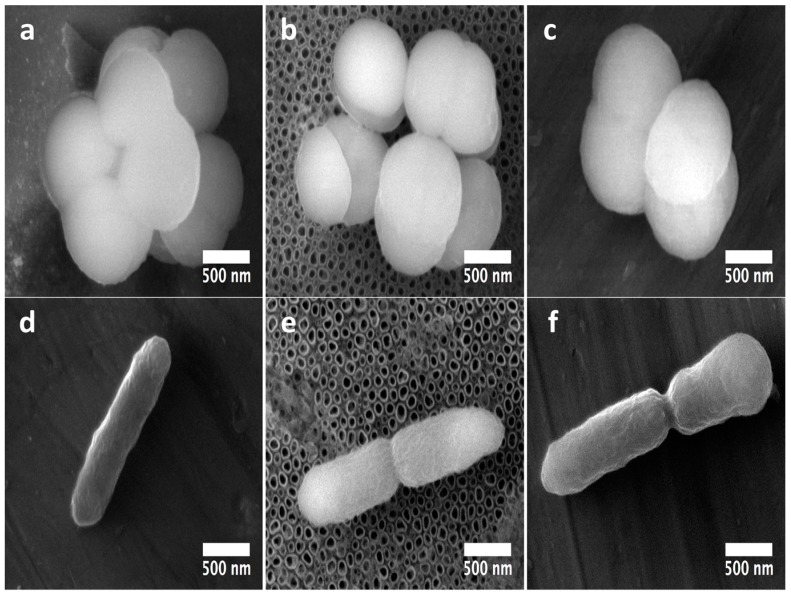
Nano-scale comparative bacteria-surface interactions among Gram-positive and Gram-negative bacteria on the experimental materials at 2 h: (**a**) *S. epidermidis* micrograph on the Flat-Ti6Al4V material; (**b**) 80 nm NTs clearly showing the poor adhesion patterns of *S. epidermidis*; (**c**) *S. epidermidis* adhesion over the rough Ti6Al4V; (**d**) *P. aeruginosa* interaction over the flat Ti6Al4V; (**e**) bacilli contact on NTs; (**f**) *P. aeruginosa* onto the Rough material.

Interestingly, staphylococci morphology at the initial adhesion phase displayed a well-defined architecture over the experimental materials (Figure 9a–c). However, on the NTs, the formation of tiny bonds directly contacting the external mouths of the NTs (Figure 9b) can be advocated, suggesting that the initial adhesion process could be disrupted by the chemical and/or morphological properties of the NTs [17]. Moreover, it can be appreciated that there is a greater number of cell-cell connections on NTs instead of cell-surface interactions; meanwhile the flat material strongly induced cellular anchorage to the material surface. Our elevated magnification zooms (with high definition) bring out important evidence that suggests promoted *S. epidermidis* anchorage on the flat material compared to the NTs. Since it has been speculated that the Gram-positive cell wall may be partially hydrophobic [23] and the Flat Ti6Al4V is believed to be the more hydrophobic experimental material, this information could explain part of the discoveries observed here. On the other hand, P. aeruginosa was more enlarged, suggesting it was better anchored on the flat material (Figure 9d). Meanwhile the bacilli morphology on the NTs was shorter with poorer cell bonding points (Figure 9e). The rough surface was detected to have a more closely related morphology to that observed on the flat material but with a smaller cell size. Despite the important roughness differences presented by the flat and rough materials, we could advocate that both surfaces are pretty similar from a chemical point of view, information which could explain part of the similar bacilli bonding and morphological behaviors detected over those surfaces.

**Figure 10:**
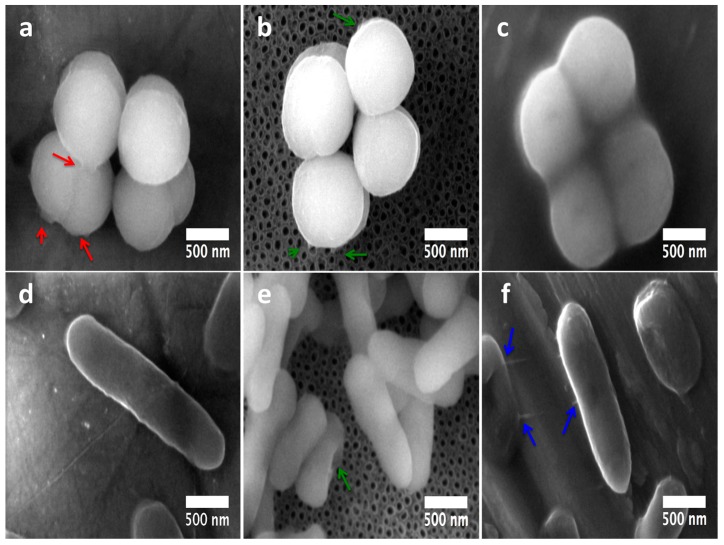
Nano-scale comparative bacteria-surface interactions among Gram-positive and Gram-negative microorganisms over the surfaces at 6 h: (**a**) *S. epidermidis* on the Flat-Ti6Al4V material (the red arrows highlight the huge contact bonds among the surface and the cell-cell interactions); (**b**) 80 nm NTs illustrating decreased adhesion of *S. epidermidis* (green arrows points out tiny surface adhesion-bonds); (**c**) *S. epidermidis* adhesion on the rough material; (**d**) *P. aeruginosa* largely disseminated on the Flat Ti6Al4V; (**e**) deformed bacilli interaction on NTs (the green arrow represents deformed morphology of direct interacting bacteria to the NTs); (**f**) *P. aeruginosa* growth on the rough material (the blue arrows show the bacterial interaction bonds above the surface).

At 6h of incubation (late adhesion), we elucidated promoted *S. epidermidis* anchorage with more defined and spread adhesion-bonds (red arrows) over the flat surface (Figure 10a). On the other hand, *S. epidermidis* showed tiny bonds (at nanoscale) strictly contacting the mouths of the NTs (Figure 10b, green arrows), proposing disturbed adhesion that connects to the nanostructured material. On the contrary, the rough surface illustrated a compact-like morphology (Figure 10c), suggesting more cell-cell attraction instead of cell-surface bonding, information that could explain the controversial decreased bacterial colonization observed in the rough material at 6h. Similarly to the flat surface behavior on *S. epidermidis*, the *P. aeruginosa* activity showed an elongated morphology, suggesting better anchorage to the material (Figure 10f). In the same way, the rough Ti6Al4V inspired a similar prolonged morphology with improved cell-surface bonds, as depicted by the blue arrows in Figure 10f. However, the nanostructured surface was able to enthuse a disturbed morphology to the bacteria that was in direct contact to the surface (Figure 10e, green arrow), leaving alone only the formation of cell-cell bindings, evidence that could explain the lower *P. aeruginosa* viability observed on the NTs (Figure 8).

Importantly, *S. epidermidis* (Gram-positive) showed decreased bacterial bonds over the NTs and a constant morphology at the early and late incubation times compared to the *P. aeruginosa* that slightly bonds to the NTs. This interesting comportment could be explained in part by the physicochemical properties of the bacterial walls. As Gram-positive bacteria have a thicker peptidoglycan layer localized at the external phase of the cell than Gram-negative bacteria [24], it may contribute to a decreased affinity to the material surfaces. Moreover, it is important to indicate that peptidoglycan is a denser polysaccharide layer (partially hydrophobic) that may not have a good ability anchor to the nanostructured layer [25]. Meanwhile, the flat, carbon-rich surface allowed better bacterial bonding. Furthermore, is vital to highlight that the staphylococci spp. presented a more opaque cell aspect with promoted cell-cell bonding compared to that of *P. aeruginosa* (which presented a translucent morphology) cultured over the experimental materials. Moreover, an increased number of bonding points for the *P. aeruginosa* was identified on the rough material at 6h compared to those of *S. epidermidis* (Figure 10c,f). However, promoted bacterial deformation (which suggest improved adhesion) was detected for the *P. aeruginosa* but not on *S. epidermidis*. This interesting trend (which has never been deeply discussed) could be due to the structural properties of the Gram-negative cell wall. Since Gram-negative microorganisms present a more fluid double membrane layer at the external phase with a thinner peptidoglycan layer (compared to Gram-positive) [26], they may allow promoted membrane anchorage with easier deformation capability (possibly by bacteria motility), which could explain the enlarged and deformed cell architecture that was formerly reported here. Although, other molecular studies such as the inhibition of bacterial receptors involved in the adhesion and mobility processes, RT-PCR, western blot, and immunofluorescence assays are recommended in order to explain these interesting interrogations.

### 2.3. Human Osteoblast Behavior

**Figure 11:**
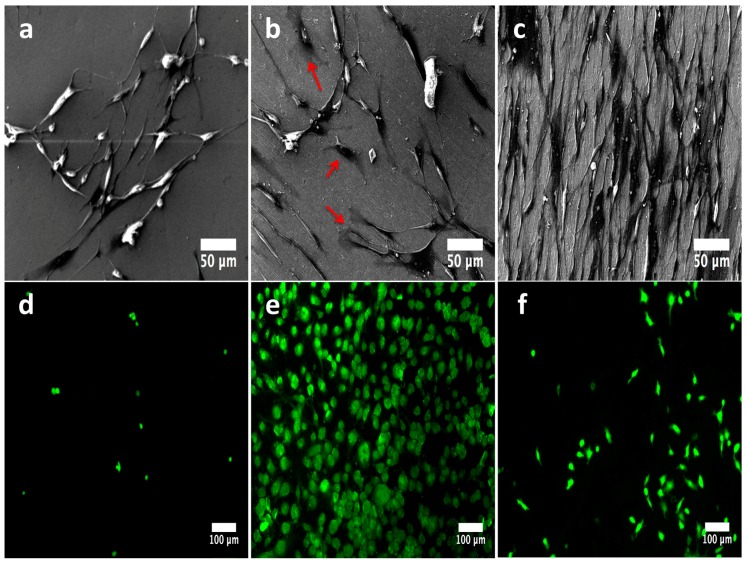
Osteoblast behavior cultured on the investigational materials after 24 h: (**a**) adhered osteoblasts on the flat surface; (**b**) 80 nm NTs illustrating promoted adhesion with long and interconnecting filopodia projections (red arrows highlight the formation of anchored sites); (**c**) cultured osteoblasts on the rough disk; (**d**) representative viable cells detected on the flat material; (**e**) vital osteoblasts analyzed on the NTs; (**f**) anchored vital osteoblasts on the rough Ti6Al4V.

Figure 11 illustrates the initial behavior of osteoblasts cultured on the experimental materials. The flat surface allowed decreased cellular anchorage with non-outcomes of well-defined cellular adhesion as there was no visible filopodia formation and a lower number of viable cells (Figure 11a,d). On the other hand, the NTs showed promoted filopodia formation with an increased amount of cell bonding (Figure 11b, red arrows), as well as a surprisingly improved number of viable cells (Figure 11e). Furthermore, the rough surface advocated an elevated number of growing cells, as evidenced by FE-SEM (Figure 11c), but with low results of adhered viable osteoblasts, as evidenced by the fluorescence staining (Figure 11f). These interesting observations suggest that flat Ti6Al4V surfaces do not favor the osteoblast anchorage; meanwhile the rough surface promotes increased levels of osteoblasts but without organized cellular growth. In addition, the NTs stimulated and promoted an ordered growing capability, information which suggests that nanostructured material could be more favorable for stimulating cellular organization with promising osteoactive capability. Our results are strongly congruent with previous works [27].

It is important to compare and highlight the main differences between the viability behavior of bacteria and osteoblasts. Interestingly, the NTs were capable of decreasing the initial adhesion step of both bacteria (Gram + and −); meanwhile, NTs were the most optimal option for osteoblasts. These interesting activities could be related to the surface nano-roughness provided by the NTs; as speculated in previous works, nanostructured materials can control at the molecular scale the deposition of proteins involved in the initial osteoblastic interactions [10,28]. In addition, nano-rough surfaces can disrupt the initial deposition of bacteria due to the substantial differences presented between the diameter of the NTs and the cell-wall size, which is bigger than the NTs, information that could explain the effects observed especially for *S. epidermidis*. Moreover, the NTs are significantly more hydrophilic than the rough and flat materials, information that suggests that NTs allow better control of osteoblatic adhesion without generating an aberrant morphology (see Figure 11). On the other hand, the improved wettability found on NTs could contribute to disturbed electrostatic interactions between the NTs and bacteria [23], mainly for *P. aeruginosa*. Table 2 presents a summarized comparison between the experimental materials and the cellular models evaluated here.

## 3. Materials and Methods

### 3.1. Ti6Al4V Surfaces Development

#### 3.1.1. Synthesis of NTs

For the manufacturing of homogenous and reproducible 80 nm diameter NTs, we performed electrochemical anodization as previously described [29,30]. Briefly, flat mirror polished (100 to 2000 grift and 1-μm alumina) disks of Ti6Al4V (ASTM F-136; Supra Alloys Inc., Camarillo, CA, USA) with 150 mm diameters and 5 mm thicknesses were assembled in a special 125 mL electrochemical cell and anodized using Microdacyn 60^®^ super-oxidized water (Oculus Technologies, Guadalajara, Jal, Mexico) at pH 6.8, supplemented with 10 mg/L of NH_4_F (Sigma-Aldrich, St. Louis, MI, USA) and 100 mg/L NaCl (Sigma-Aldrich, USA). Next, a potential of 20 V was applied for 5 min at room temperature (RT) using a DC power supply and a platinum mesh as a counter electrode. Afterwards, the samples were cleaned for 5 min under sonication, rinsed with isopropyl alcohol, and desiccated for 12 h. Importantly, all experimental specimens were sterilized using UV irradiation (285 nm UVB light source) for 30 min each side inside of a class II biosecurity cabinet. Finally, a sterilized flat mirror polished Ti6Al4V disk was used as a control for all the experimental assays.

#### 3.1.2. Rough Ti6Al4V Surface

In order to increase the surface roughness of the Ti6Al4V substrates, we performed mechanical polishing following a metallographic procedure using SiC emery paper (from 100 to only 400 grift) (part of the procedure described in ASTM E3-11). Next, the samples were ultrasonically washed in acetone, ethanol, and water (three times for 5 min each), with the purpose of removing any abrasive particle generated by the mechanical polishing. Finishing, the samples were dried into a desiccator for 24 h and sterilized as described above.

### 3.2. Surface Physicochemical Characterization

#### 3.2.1. FE-SEM Analysis of the Experimental Materials

As an endeavor to characterize the surface morphology of the samples, we applied FE-SEM (Tescan LYRA 3, Brno, Czech Republic), taking micrographs at 20 kV accelerating voltage on diverse random fields.

#### 3.2.2. Chemical Characterization

The chemical assays were performed using EDX (Tescan LYRA 3, Tescan, Brno, Czech Republic), with a silicon drift detector coupled to the FE-SEM.

#### 3.2.3. Roughness Evaluation

In contemplation of the material´s surface roughness, we performed AFM (Quesant Q-Scope 350, AMBIOS, Agura Hills, CA, USA), equipped with an anti-acoustic box to prevent noise, which can affect the measurements. The operation scan rate was 1 Hz by contact mode at RT. A 40-μm X-Y and 4-μm Z, a scanner equipped with a silicon tip and 10 nm tip curvature was used. The scan surface area was 1 μm^2^. For quantitative assessment of the roughness topographies, we provide an analytical analysis of the arithmetic average (Ra).

### 3.3. Bacterial Behavior among the Experimental Surfaces

#### 3.3.1. Bacterial Culture

For the bacterial-surface interaction analyses *S. epidermidis* (ATCC 12228) and *P. aeruginosa* (ATCC 27853) (microbial models of orthopedic importance) were used [7,31]. For the formulation of the inoculums, both strains were freshly grown overnight on tryptic soy agar (TSA) plates (Beckton Dickinson, Sparks, MD, USA). Discrete colonies of each bacteria were obtained from TSA plates and separately suspended in tryptic soy broth (TSB) to an optical density (O.D.) of 0.05 (*S. epidermidis*) and 0.04 (*P. aeruginosa*) with pH 7.0; assessed using a spectrophotometer (LAMBDA 25, Perkin Elmer, CT, USA).

#### 3.3.2. Bacterial Adhesion and Viability Assays

For the assessment of the bacterial behavior on the materials, 100 μL of each microbial suspension containing approximately 1 × 10^7^ CFU/mL (O.D. 0.05 and 0.04) plus 100 μL of warm TSB was used to fully cover the surface and avoid sample desiccation. The inoculums were incubated onto the specimens for 2 and 6 h at 37 °C in a static model. After each incubation time, the surfaces were washed three times in 1X PBS for 5 min to discard any unanchored bacteria. Next, each material model was deposited into an individual well of a sterile 24-well polystyrene plate (Corning, Corning, NY, USA) containing 1 mL of fresh TSB. The plate was positioned in an ultrasonic bath (Branson, Branson, MO, USA) and sonicated at 120 W for 1 min at periods of 5 s to evade cellular lyses. Thereafter, the surfaces were scraped off using a sterile surgical blade to entirely detach any bounded cell. The materials were taken off and the remaining suspensions were diluted with Phosphate Buffer Saline (PBS) and cultured at 37 °C for 24 h in TSA and counted for analytical assessment.

#### 3.3.3. Bacterial Approach by FE-SEM

Bacterial morphology is of importance to understand the mechanisms regarding bacterial adhesion and bonding anchorage to materials’ surfaces [23]. Moreover, the colonization rate is vital to assess the bacterial behavior over biomaterials, so, in order to address these critical parameters, we performed FE-SEM on each material at 2 h (indicated as initial adhesion phase) and 6 h (late adhesion phase) of incubation. Briefly, each sample was rinsed with PBS three times for 5 min respectively, fixed with 2.5% glutaraldehyde for 2 h at RT and dehydrated in graded series of ethanol solutions (30 min each). As an endeavor to detail and directly visualize the bacterial morphology and the cellular bonding on the surface, we applied high resolution FE-SEM with a very high magnification scale [29,32]. Fixed cells were not sputter-coated in order to avoid the coverage of the nano-bonding phase at a very high amplification. The zoom applied was 100,000×, operating at 10 kV and a working distance of 6 mm.

### 3.4. Human Osteoblasts Characterization over the Materials

#### 3.4.1. Osteoblasts Culture

Human osteoblasts were cultured using Dulbecco´s modified Eagle’s Medium (DMEM; Gibco-Invitrogen, Carlsbad, CA, USA), supplemented with 10% fetal bovine serum (FBS; Gibco-Invitrogen) and 1% penicillin/streptomycin (Gibco-Invitrogen) at 37 °C in 5% CO_2_. Each experimental sample was positioned in an individual well of a 12-well polystyrene plate (Corning, NY, USA). Next, the cells were cultured using 1 mL of medium containing a concentration of 2 × 10^4^ cells per mL of the materials and stored in a CO_2_ chamber for 24 h [10].

#### 3.4.2. Cellular Viability by Fluorescence Microscopy

Osteoblast viability after 24 h of incubation was explored among the experimental substrates using a live/dead viability/cytotoxicity assay kit (Gibco-Invitrogen, Carlsbad, CA, USA), mixing 1mM calcein-AM and 2 mg/mL of ethidium homodimer-1 following the manufacturer’s instructions. Afterwards, the materials were inverted and mounted onto cover slides with a fluorescence mounting medium (Sigma-Aldrich, USA), characterized and photographed with a green (live) and red (dead) filter under an inverted fluorescence microscope (ZOE, Bio-Rad, Hercules, CA, USA). At least five fields were randomly imaged. 

#### 3.4.3. Osteoblast Morphology by FE-SEM

In order to characterize the early cellular morphology among the tested surfaces after 24 h of culture, we performed FE-SEM. The cells were washed, fixed, and dehydrated as described in Section 3.3.

## 4. Conclusions

The present study assess for the first time the evaluation, comparison, and discussion of flat, nanostructured, and rough Ti6Al4V surfaces on the behavior of bacterial adhesion of two important orthopedic bacterial models. Moreover, the important structural and viable properties detected between Gram-positive and Gram-negative bacteria (discussed for the first time here) used here were pointed out. Our results reveal that chemical differences among the material surfaces can modulate strong alterations in bacterial activity with critical change to the cellular architecture, allowing the formation of promoted or reduced bacteria-surface interactions, instead of only surface roughness properties. Moreover, the nanostructured surface advocated the lower bacterial viability and the reduced cell adhesion capability with decreased results of colonization movement. Importantly, the nanostructured surface stimulates a superior osteoblast organization with elevated filopodia formation to the flat and even rough material, proposing the nanostructured Ti6Al4V surfaces as optimal for bone implant applications. The NTs were able to alter the ideal bacterial activity over Gram-positive and Gram-negative materials. Our results open up a novel pathway for the investigation and comparison of the effects of Gram-positive and Gram-negative cell walls on the behavior of biocompatible surfaces for the understanding of implant-related infections.

## Figures and Tables

**Table 1:** Chemical composition of the materials surfaces by energy dispersive X-ray spectroscopy (EDX).

Sample	C	V	Al	Ti	O	F
Flat	3.22	4.12	5.60	87.06	-	-
NTs	1.33	-	4.12	68.38	23.46	2.71
Rough	3.99	3.89	6.70	79.44	5.98	-

**Table 2:** Summarized comparison between the physicochemical surface properties and the optimum surface option for cellular behavior. ‘x’ represents low viability, ‘xx’ indicates regular viability, and ‘xxx’ strong viability. ‘*’ expressed low adhesion, ‘**’ displayd regular adhesion, and ‘***’ announces high adhesion.

	Flat	NTs (Nano-Rough)	Rough
*S. epidermidis*	xx ***	x *	xxx **
*P. aeruginosa*	xx **	x *	xx **
Osteoblasts	x	xxx	x
